# Neuromuscular electrical stimulation in garments optimized for compliance

**DOI:** 10.1007/s00421-023-05181-9

**Published:** 2023-04-03

**Authors:** R. Juthberg, J. Flodin, L. Guo, S. Rodriguez, N. K. Persson, P. W. Ackermann

**Affiliations:** 1 Department of Molecular Medicine and Surgery, Karolinska Institutet, Stockholm, Sweden; 2 Smart Textiles and Polymeric E-textiles, Swedish School of Textiles, University of Borås, Borås, Sweden; 3 School of Electrical Engineering and Computer Science, KTH Royal Institute of Technology, Stockholm, Sweden; 4 Department of Trauma, Acute Surgery and Orthopaedics, Karolinska University Hospital, Stockholm, Sweden

**Keywords:** Electric stimulation therapy, Pain, Patient comfort, Patient compliance

## Abstract

**Purpose:**

Physical inactivity is associated with muscle atrophy and venous thromboembolism, which may be prevented by neuromuscular electrical stimulation (NMES). This study aimed to investigate the effect on *discomfort, current amplitude* and *energy consumption* when varying the frequency and phase duration of low-intensity NMES (LI-NMES) via a sock with knitting-integrated transverse textile electrodes (TTE).

**Methods:**

On eleven healthy participants (four females), calf-NMES via a TTE sock was applied with increasing intensity (mA) until ankle-plantar flexion at which point outcomes were compared when testing frequencies 1, 3, 10 and 36 Hz and phase durations 75, 150, 200, 300 and 400 µs. Discomfort was assessed with a numerical rating scale (NRS, 0–10) and energy consumption was calculated and expressed in milli-Joule (mJ). Significance set to *p* ﻿≤ 0.05.

**Results:**

1 Hz yielded a median (inter-quartile range) NRS of 2.4 (1.0–3.4), significantly lower than both 3 Hz with NRS 2.8 (1.8–4.2), and 10 Hz with NRS 3.4 (1.4–5.4) (both *p* ≤ .014). Each increase in tested frequency resulted in significantly higher energy consumption, e.g. 0.6 mJ (0.5–0.8) for 1 Hz vs 14.9 mJ (12.3–21.2) for 36 Hz (*p* = .003). Longer phase durations had no significant effect on discomfort despite generally requiring significantly lower current amplitudes. Phase durations 150, 200 and 400 µs required significantly lower energy consumption compared to 75 µs (all *p* ≤ .037).

**Conclusion:**

LI-NMES applied via a TTE sock produces a relevant plantar flexion of the ankle with the best comfort and lowest energy consumption using 1 Hz and phase durations 150, 200 or 400 µs.

## Introduction

Physical inactivity in the form of postoperative immobilization is highly associated with muscle atrophy and an increased risk of venous thromboembolism (VTE) (Abeles et al. 2017; Van Ancum et al. 2017). Intermittent pneumatic compression (IPC) of the lower limbs is an evidence-based technique used for VTE prophylaxis (Anderson et al. 2019; Kakkos et al. 2016; Pavon et al. 2016). However, IPC has not been shown to reduce muscle atrophy and has compliance issues when used as VTE prophylaxis outside hospitals, due to its non-mobile nature (Faghri et al. 1997; Froimson et al. 2009; Kahn and Ginsberg 2002; Masri et al. 2004; Urbankova et al. 2005).

Therefore, neuromuscular electrical stimulation (NMES) has emerged as an alternative mobile technique to produce muscle contraction of e.g. the calf. When the calf-NMES contraction induces plantar flexion of the ankle this significantly improves venous return (Broderick et al. 2014; Praxitelous et al. 2018), which has also been shown to reduce the risk of VTE (Hajibandeh et al. 2017). However, the main problem with standard NMES is poor patient compliance, mainly due to the repetitive application of gel electrodes that should be placed on individual motor points, which furthermore have to be found manually by the user (Maffiuletti et al. 2018).

This has led to the development of integrating NMES into textiles, which have the potential to increase patient compliance. Previous studies have demonstrated that textile electrodes integrated into socks are well suited for NMES (Crema et al. 2018; Gniotek et al. 2011) and therefore we created a sock with integrated *transverse textile electrodes* (TTE), with the electrodes positioned to overlay motor point locations as suggested by Botter et al. (2011). However, the optimal NMES parameter settings for high comfort and low energy consumption using textile electrodes are unknown.

The higher frequencies within the range of 1–50 Hz, when using standard gel electrodes, have been suggested to reduce the current amplitudes required to produce muscle contraction (Baker et al. 1993; Flodin et al. 2022; Gobbo et al. 2014; Mettler et al. 2018). Previous studies which included evaluation of comfort have suggested a frequency of around 36 Hz to be comfortable while at the same time not causing excessive muscle fatigue (Baker et al. 1993; Breen et al. 2012; Broderick et al. 2014; Lyons et al. 2002). In addition, several studies have indicated that longer phase durations are associated with better patient comfort (Bowman and Baker 1985; Scott et al. 2014, 2009). However, all the above studies utilized standard non-textile electrodes to administer such high-intensity NMES that the threshold for muscle contraction was surpassed to produce moderate- to high-level contractions relative to the maximal voluntary contraction. Whether calf stimulation with low-intensity (LI) NMES, (i.e. minimal NMES intensity that can induce a clinically relevant muscle contraction) via TTE yields similar frequency- and phase duration-associated effects as higher intensity NMES using standard electrodes is not known. Another aspect that appears not to be well studied is what frequencies and phase durations that are associated with the minimal energy required per induced muscle contraction.

Thus, the aim of this study was to investigate the effect of frequency and phase duration, on discomfort, current amplitude and energy required to induce a plantar flexion of the ankle, when LI-NMES was administered via TTE integrated into a sock. The primary hypothesis was that variations in frequency and phase duration yield similar outcomes when using LI-NMES with TTE as have been demonstrated in previous studies using higher intensity NMES with standard electrodes. The secondary hypothesis was that the lowest tested LI-NMES frequency (1 Hz) and the shortest phase duration (75 µs) would require the least amount of energy.

## Methods

### Participants and study design

This was an explorative study including eleven healthy participants. The study investigated the effect of different NMES parameter settings on required current amplitude, energy consumption and discomfort. Inclusion criteria were age ≥ 18 and voluntary participation. Exclusion criteria were pregnancy, pacemaker, ongoing thromboprophylactic treatment or, skin wounds, vascular abnormalities or previous vascular system surgery, in the lower limbs. All participants were asked to sign an informed consent and to fill in information about themselves, including sex, age, height, weight and estimated physical activity (1–6) according to the Grimby/Frändin activity scale (Grimby and Frändin 2018) (Table 1).

**Table 1:** Demographics and characteristics of the participants

Participant	Sex	Age, years	Height, cm	Weight, kg	Physical activity level^a^
1	Female	24	173	55	3
2	Male	47	180	80	5
3	Female	23	173	70	3
4	Male	55	164	62	5
5	Male	25	187	73	3
6	Male	29	187	100	4
7	Female	57	167	57	5
8	Male	37	180	86	5
9	Male	26	174	57	6
10	Male	27	170	60	3
11	Female	23	165	56	3
Median (Inter-quartile range)		27 (24–47)	173 (167–180)	62 (57–80)	4 (3–5)

^a^Frändin/Grimby activity scale (1–6)

### Materials

**Fig. 1:**
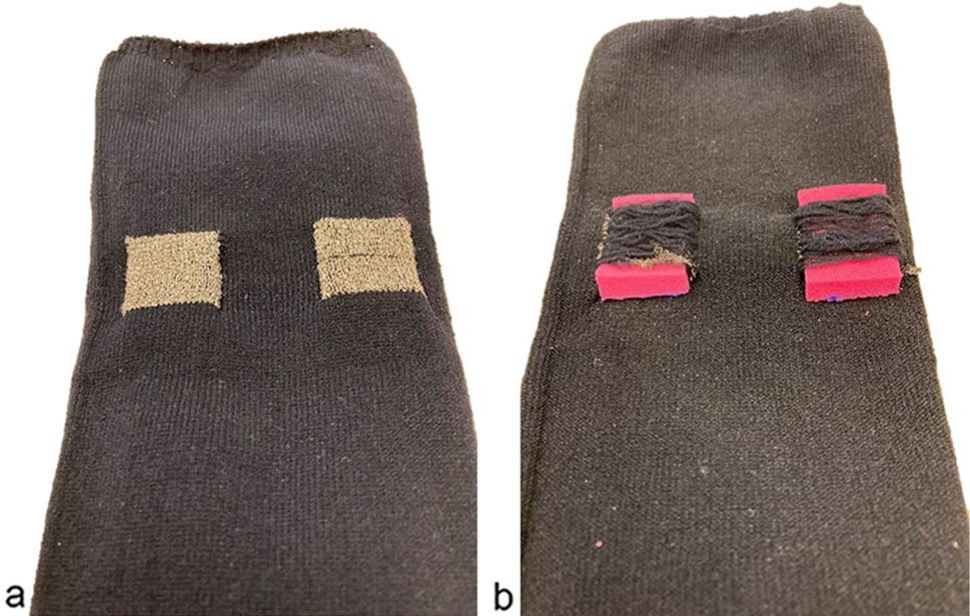
**a**–**b** Transverse textile electrodes (TTE) **a** Inside aspect of sock displaying TTE surface **b** Outside aspect of sock displaying TTE with overlaying pink melamine sponges held in place with polyamide/Lycra elastic yarn floats

**Fig. 2:**
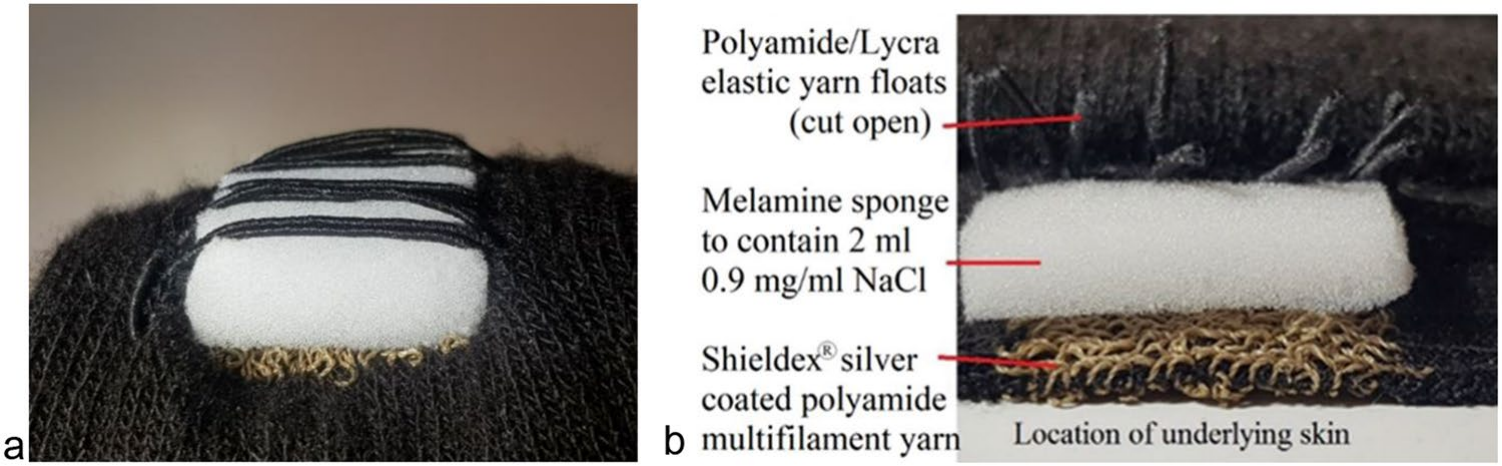
**a**–**b** Layers of the transverse textile electrodes (TTE) **a** A longitudinal view of an uncut TTE **b** A transversal view of a longitudinal cut-through section displaying the layers of the TTE

A prototype-sock was specifically constructed for this study, using a Shima Seiki whole garment flat knitting machine (Shima Seiki MFG Ltd, Japan), utilizing an electrode setup designated TTE, short for *transverse textile electrode(-s)*. The TTE setup consisted of two rectangular textile electrodes (2 × 2.5 cm) transversely knitted with the inner edges 1.8 cm apart into the back of a sock (polyamide/Lycra blended yarn, Fig. 1). The electrodes were placed approximately at the largest circumference of the calf with the electrodes positioned to cover areas of the calf which according to a motor point map created by Botter et al. (2011) in general has a high likelihood of containing a motor point. When the sock was applied to the calf, each textile electrode stretched to the size of approximately 3 × 3 cm, and the distance between the inner edges of the textile electrodes increased to approximately 3 cm. The electrodes and the socks were simultaneously knitted in a single process using intarsia knitting which allows for the seamless integration of patterns of functional components. The material of the electrodes was silver-coated polyamide multifilament yarn, with the trade name Shieldex® (produced by Statex Produktions und Vertriebs GmbH). On the outside of the sock, there were elastic yarn floats (polyamide/Lycra) spanning mediolaterally over each textile electrode to hold a regular melamine cleaning sponge in direct contact with the textile electrode (0.5 × 3 × 3 cm). The purpose of this was to increase local pressure and contact and to enable moisturization (2 ml 0.9 mg/ml NaCl per sponge) of the electrode/skin interface to facilitate electrical conduction (Euler et al. 2021). Finally, crocodile clips were used to connect the outside of the electrode fabric of the TTEs to the wires from the constant current NMES device (Chattanooga Physio, DJO Nordic, Malmoe, Sweden). The different layers of the textile electrode are displayed in Fig. 2.

### Measurement of outcomes

The overall purpose of this study was to find ways to minimize discomfort and energy consumption, to increase compliance with future iterations of garment-integrated NMES. Because of this, a concept was applied of keeping current amplitude to a minimum to reduce energy consumption, while still having a clinical effect. To meet both these criteria we choose plantar flexion of the ankle induced by the lowest possible milliampere (mA) current amplitude of calf NMES as the point of measurement for outcomes. In this text, we refer to the concept described above of low current amplitude NMES as low-intensity (LI) NMES, while the concept of low overall energy consumption NMES is referred to as low-energy (LE) NMES. Plantar flexion of the ankle induced by LI-NMES has in a previous study been shown to increase venous blood flow while still being comfortable (Laverick et al. 1990). LI-NMES has also been suggested to be clinically sufficient for endurance training among patients who have suffered disuse atrophy or who are unable to handle the much less comfortable concept of maximal contraction NMES (Baker et al. 1993). Beyond this, the choice of a visible plantar flexion of the ankle as a threshold for measurement of outcome is beneficial because of its simple and dichotomous nature, the plantar flexion is either visible or not. The method also has the added benefit of being easy to reproduce without any special equipment.

For all tests in this study, participants were seated in a regular chair with their knees flexed approximately 90 degrees. The leg side was randomized, and the TTE sock was applied to the calf of the randomized leg. Testing started by applying the lowest possible mA current amplitude followed by as small as possible stepwise increases until one of three outcomes occurred; (1) a NMES induced plantar flexion of the ankle became visible to the examiner, (2) the test was aborted by the examiner due to insufficient electrical connection, or (3) the test was aborted by the participant. If plantar flexion was achieved, measurements of the outcome’s *current amplitude, energy consumption* and *discomfort,* were performed at this point.

An estimation of *discomfort* was made by the participant at plantar flexion using a numerical rating scale (NRS) ranging from 0 to 10, where 0 corresponded to no discomfort and 10 corresponded to the worst imaginable discomfort. Participants were instructed to estimate NRS from 0 to 10 in steps of 1.

The outcome *current amplitude* was defined as the minimal LI-NMES current amplitude in milliampere (mA) required to induce a visible plantar flexion of the ankle.

The outcome *energy consumption* was defined as the minimal NMES energy in milliJoule (mJ) per stimulation cycle required to induce a visible plantar flexion of the ankle. The NMES stimulation cycle consists of the ON-time, where stimulation pulses occur, and the OFF-time, where stimulation pulses do not occur. The ON-time is often divided into three distinct parts; (1) the ramp-up time during which pulse amplitudes are gradually increased until stabilizing in (2) the plateau time, during which amplitudes are held constant at a plateau until the transition into (3) the ramp-down time, during which pulse amplitudes are gradually decreased to zero.

**Fig. 3:**
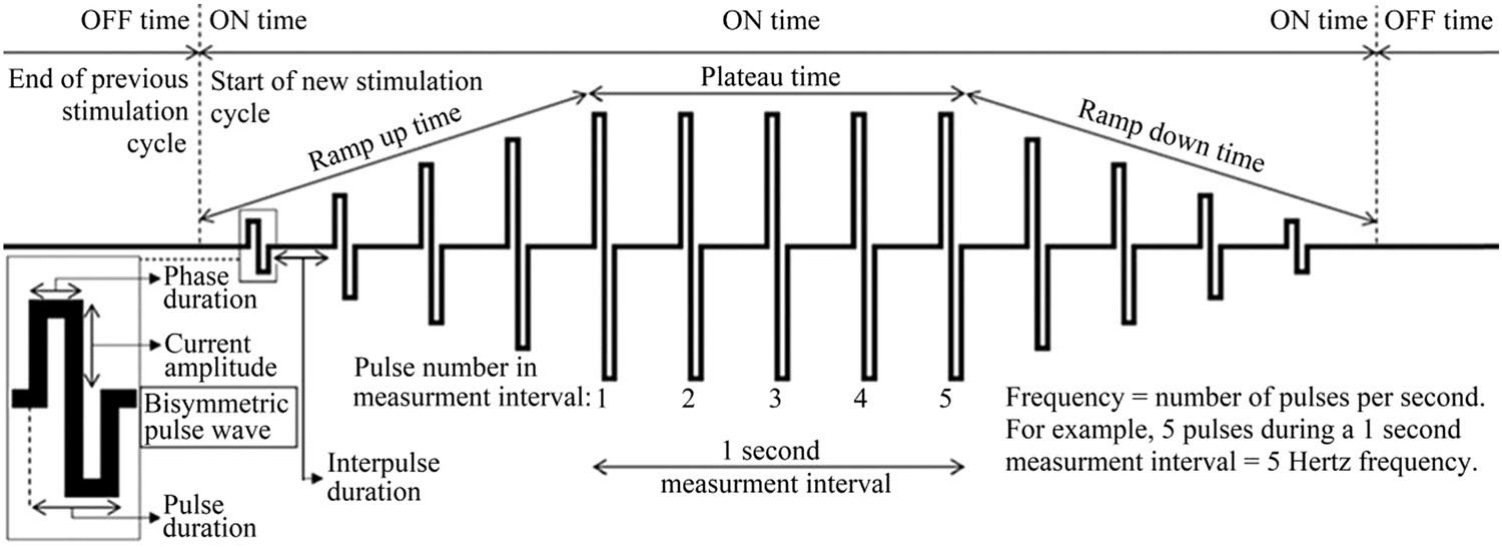
Examples of parameter settings that may be altered to minimize current requirements and discomfort during ankle plantarflexion induced by neuromuscular electrical stimulation

Normally this is the end of the ON time, which is followed by the OFF time, a period of electrical silence lasting until the start of the next stimulation cycle. The ratio between the ON time and the OFF time (“duty-ratio”) is most often set so that the OFF time is longer than the ON time to give the muscle time to reset after each contraction. There are many different types of pulses, but the one used in this study, the bisymmetric (short for biphasic symmetric) rectangular wave pulse, consists of a rectangular positive phase of a certain duration and amplitude, which is immediately followed by a negative phase of the same duration and absolute amplitude (Fig. 3). All of these parameter settings may affect the energy consumption.

For this study, there was no access to equipment able to continuously measure the total resistance of the skin and the skin–electrode interface during stimulation. However, several separate measurements of total resistance between the electrodes of the TTE with the moisturized sponges averaged out to approximately 1 kΩ, and this value was assigned to all participants to be able to calculate the energy consumption per stimulation cycle at plantar flexion. This value was also in line with suggested resistance values of 1 – 100 kΩ for skin with varying degrees of moisturization (Fish and Geddes 2009). The formula for the calculation of the energy consumption per NMES stimulation cycle required to induce plantar flexion is presented below.

$$E_{{{\text{total}}}} = \left( {\frac{RUT + RDT}{2}} \right) + PT*F*PD*(MCA)^{2} *R$$

Abbreviations used in energy calculation formula (unit used in energy calculation formula)

E_total_ = Total energy consumption per NMES stimulation cycle (Joule)

*RUT* = Ramp-up time (seconds)

*RDT* = Ramp-down time (seconds)

*PT* = Plateau time (seconds)

*F* = Frequency (Hertz)

*PD* = Pulse duration (= 2 * phase duration) (seconds)

*MCA* = Max current amplitude (Ampere)

*R* = Resistance (Ohm).

### NMES parameter nomenclature

In our opinion, there seems to be some confusion regarding the nomenclature and meaning of the term “pulse duration” when using the bisymmetric rectangular waveform (Fig. 1). For example, when setting the “pulse duration” of the Chattanooga Physio NMES device used in this study, what actually was set was the duration of each of the positive and negative phase durations of the bisymmetric rectangular wave, as corroborated by internal bench testing of the device´s actual phase and pulse durations. Therefore, the setting that according to the Chattanooga Physio NMES device manual, and seemingly many previous studies on NMES, is referred to as the “pulse duration”, is in this text referred to as the phase duration. This is in accordance with the nomenclature recommended by Baker et al. (1993), which stated “*With biphasic pulses, pulse duration is technically the duration of the entire waveform, including both the positive and the negative phases. However, it is the duration of each phase that is important in determining excitability. It is, therefore, important to specify phase duration rather than pulse duration when describing symmetric biphasic pulses.*” When possible, all references in this text using the expression “pulse duration” have been checked to ensure that they can be referred to as “phase duration”, according to the definition above.

### Parameter settings

The study on calf-NMES via TTE (bisymmetric rectangular wave, 0.5 s ramp-up time, 1.5 s plateau-time, 0.5 s ramp-down time, 6 s OFF-time) examined the effect of variations in frequencies (1, 3, 10 and 36 Hz) and phase durations (75, 150, 200, 300 and 400 µs) on discomfort, current amplitude and energy consumption. Beyond the choice to include 36 Hz based on previous NMES comfort studies (Breen et al. 2012; Broderick et al. 2014; Lyons et al. 2002), the lower frequencies (1, 3 and 10 Hz) as well as a wider range of phase durations were included to study alternatives that might have a lower overall energy consumption.

### Statistics

Due to the relatively low number of participants and the nature of the outcome measures, non-parametric statistics were used for all analysis. Descriptive statistics for participant characteristics were presented with median and min–max range while inferential statistics were presented with median and inter-quartile range (IQR). Wilcoxon signed-rank test was used to calculate differences between outcomes, which were considered significant if *p* ≤ 0.05. All data were analyzed using SPSS version 28 (IBM Corp. Released 2016. IBM SPSS Statistics for Windows, Armonk, NY: IBM Corp.)

Because the study investigated four different frequencies (1, 3, 10 and 36 Hz) and five different phase durations (75, 150, 200, 300 and 400 µs), data were collected for a total of 20 different combinations enabling for a total of 190 possible related samples comparisons. To limit this number, comparisons were only made between frequencies (six comparisons) and between phase durations (ten comparisons). This was achieved by averaging out the outcome scores for the parameter not in focus. For example, when extracting the participant median NRS for frequency 1 Hz, for each participant, a unique NRS score was registered for each and every investigated phase duration during 1 Hz NMES and averaged out to create a set of values, where each value represented the NRS score at 1 Hz for one participant (representative of all phase durations). Then, non-parametric statistics were applied to this set of values from which the participant median NRS for frequency 1 Hz was extracted and compared to NRS scores for other frequencies obtained in the same way.

## Results

### Frequency-dependent effects on discomfort, current amplitude and energy consumption using calf LI-NMES with TTE

The lowest median NRS score of 2.0 and IQR (0.6–5.2) resulted from LI-NMES with TTE administered with a 36 Hz frequency, however, without any significant difference to the other frequencies. Instead, 1 Hz was shown to induce significantly less discomfort with NRS 2.4 (1.0–3.0) as compared to both 3 Hz with NRS 2.8 (1.8–4.2, *p* = 0.007), and 10 Hz with NRS 3.4 (1.4–5.4, *p* = 0.014) (Table 2).

**Table 2:**
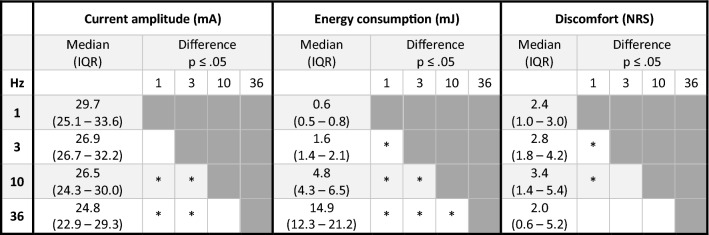
Effect of frequency on current amplitude, energy consumption and discomfort

Outcomes of calf LI-NMES administered via TTE for frequencies 1, 3, 10 and 36 Hz, expressed with median and IQR. *n* = 11. Column and row crossings marked with * indicate a significant difference between those column/row frequencies at *p* ≤ .05 according to Wilcoxon signed-rank test. *mA*  milli-Ampere, *mJ*  milli-Joule, *NRS* numerical rating scale, *IQR* interquartile range, *Hz*  Hertz, *Calf LI-NMES *low-intensity neuromuscular electrical stimulation of the calf, *TTE *transverse textile electrode

When investigating the effect of different frequencies (1, 3, 10 and 36 Hz) when using calf LI-NMES, it was shown that every increase in frequency was correlated to a gradually lower current amplitude required to induce a plantar flexion. Thus, LI-NMES with a frequency of 36 Hz resulted in the lowest median current amplitude required to induce plantar flexion (24.8 mA), significantly lower than for both 1 Hz (29.7 mA, *p* = 0.010) and 3 Hz (26.9 mA, *p* = 0.010), and with a non-significant difference compared to 10 Hz (26.5 mA, *p* = 0.062) (Table 2).

However, when looking at energy consumption, LI-NMES produced frequency-dependent increases in energy required to induce a plantar flexion, where 1 Hz was significantly more energy efficient at producing a plantar flexion compared to all other frequencies (0.6 mJ vs. 1.6–14.9 mJ, all *p* ≤ 0.003) (Table 2).

### Phase duration effects on current amplitude, energy consumption and discomfort using calf LI-NMES with TTE

Longer phase durations (75–400 us) were associated with lower current amplitudes (mA) needed to induce a plantar flexion with calf LI-NMES using TTE. All phase durations exhibited significant differences in mA (all *p* ≤ 0.003), except between 200 and 300 µs, where 400 µs required the lowest median current amplitude (17.3 mA) to induce a plantar flexion.

The low required current amplitude when using 400 µs contributed to this parameter setting also being one of the most energy efficient as it required a median of 210 mJ per NMES-cycle to induce a plantar flexion, significantly better than 75 µs (297 mJ, *p* = 0.004) and 300 µs (244 mJ, *p* = 0.021). However, the lowest median mJ values were achieved when using the 200 µs (192 mJ) and 150 µs (208 mJ), which both were significantly better than 75 µs (both *p* ≤ 0.037) (Table 3). The different phase durations did not produce significant differences in discomfort as assessed with NRS.

**Table 3:**
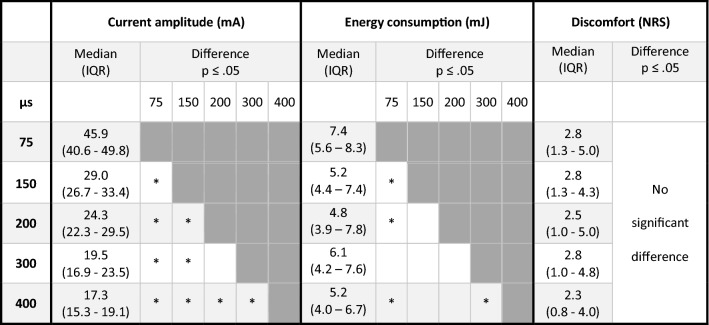
Effect of phase duration on current amplitude, energy consumption and discomfort

Outcomes of calf LI-NMES administered via TTE for phase durations 75, 150, 200, 300 and 400 µs, expressed with median and IQR. *n* = 11. Column and row crossings marked with *indicate a significant difference between those column/row phase durations at *p* ≤ .05 according to Wilcoxon signed-rank test. *mA *milli-Ampere, *mJ *milli-Joule, *NRS *numerical rating scale, *IQR *interquartile range, *Hz *Hertz, *Calf LI-NMES *low-intensity neuromuscular electrical stimulation of the calf, *TTE *transverse textile electrode

## Discussion

This explorative study demonstrated that variations in frequency (1–36 Hz) during calf LI-NMES via TTE can yield significant differences regarding current amplitude, energy consumption and discomfort. However, although variations in pulse duration (75–400 µs) yielded significant differences in current amplitude and energy consumption, there were no significant differences regarding discomfort. The least amount of discomfort was experienced at the lowest and highest frequencies tested (1 and 36 Hz). A stepwise increase of the tested frequencies required gradually lower current amplitudes, while simultaneously having a gradually higher energy consumption when inducing a plantar flexion. Longer phase durations required gradually lower current amplitudes to induce a plantar flexion, but the current amplitudes didn´t seem to affect the discomfort. The lowest median energy required per stimulation cycle to induce a plantar flexion occurred when using a 200 µs phase duration, this was, however, only significantly better than when using a 75 µs phase duration.

The main finding of this study was that when inducing a plantar flexion of the ankle with LI-NMES via TTE integrated into a sock, the highest and lowest tested frequencies of 1 and 36 Hz generated the least amount of estimated NRS for discomfort. The finding that 36 Hz yielded the lowest median discomfort is in line with suggestions used in previous studies (Breen et al. 2012; Broderick et al. 2014; Lyons et al. 2002) using higher-intensity NMES. However, NRS at 36 Hz was not significantly lower than for any other tested frequency. Instead, NRS reported for 1 Hz, was significantly lower compared to both 3 and 10 Hz. This is to the best of our knowledge novel and might be attributed to the use of LI-NMES. The higher comfort observed with 1 and 36 Hz implies that these frequencies could be used to achieve a higher compliance to calf LI-NMES administered via TTE.

The subsequent choice between 1 Hz or 36 Hz would then be dependent on other factors such as current amplitude and energy consumption. Our study found that 1 Hz was significantly more energy efficient compared to all other frequencies, in line with our secondary hypothesis. This suggests that if energy consumption is considered the most important factor, e.g. in a mobile NMES-device which preferably would incorporate the smallest battery possible, a 1 Hz frequency would be the best choice. However, if a lower current amplitude is preferred, e.g. in any NMES-device where the battery capacity is not a factor of concern, then 36 Hz may be chosen.

The observations in this study that calf NMES exhibited a frequency- and phase duration-dependent decrease in current amplitude required to induce a plantar flexion is novel when specifically looking at LI-NMES administered via TTE integrated into a sock. However, the findings are in line with previous studies using higher intensity NMES via standard electrodes and was in that regard expected (Baker et al. 1993; Flodin et al. 2022; Gobbo et al. 2014; Mettler et al. 2018), and in accordance with our primary hypothesis.

The main reason for using plantar flexion as the deciding factor for a reliable calf contraction of the LI-NMES was two-fold. First, plantar flexion is a binary outcome, either you have it or not, i.e., the test is easily reproducible with fairly high reliability. Secondly, earlier studies have indicated that plantar flexion produces a significant increase in venous return (Broderick et al. 2014; Laverick et al. 1990), which is equal to the degree of venous blood flow produced by IPC devices that are clinically used to prevent VTE in immobilized patients (Anderson et al. 2019; Kakkos et al. 2016; Pavon et al. 2016; Praxitelous et al. 2018; Urbankova et al. 2005). One major benefit of the NMES technique over IPC is that it utilizes a form of energy harvesting that reduces the external energy required for the system to induce a plantar flexion (Roy et al. 2022). The energy required to induce a plantar flexion, roughly estimated to be 200 mJ, is significantly more than the maximum energy input from the NMES stimulation registered in this study of 21.2 mJ. Given such proposed concept, an NMES-driven TTE sock may act as an energy harvesting system.

Increasing venous return during immobility may not only be useful for immobilized patients but may also apply for an aging population with decreasing activity as well as for people with sedentary work in offices-spaces with prolonged sitting. A recent study showed that persons who are the least physically active also are the ones who have the highest risk of all-cause and cardiovascular mortality if exposed to prolonged sitting (Stamatakis et al. 2019). Because of this, our study tested a concept of garment-integrated TTE LI-NMES treatment, which in the future may become an integral element of daily living that can help reduce the risk of morbidity- and mortality related to prolonged immobility and sitting.

The second main finding of this study was that there were no significant differences in discomfort between the different phase durations examined. This finding is in contrast with two earlier studies which demonstrated that high-intensity NMES of the quadriceps was better tolerated when applying longer phase durations, 500 µs was significantly better than 200 µs, which was significantly better than 50 µs (Bowman and Baker 1985; Scott et al. 2014, 2009). The observed discrepancy compared to our study, may be explained by the use of LI-NMES which produces less discomfort as compared to the higher intensity NMES used in the two earlier studies.

The observation that phase duration during LI-NMES does not affect discomfort suggests that the decision of which phase duration to use will be dependent on other factors including current amplitude and energy consumption. The finding that there were non-significant differences in energy consumption between the three best phase durations (150, 200 and 400 µs), suggests that in a situation where energy consumption is preferred, such as in a mobile NMES device with a lower battery capacity, any one of these phase durations may be chosen.

When gradually testing longer phase durations (75–400 µs) during calf LI-NMES, significant stepwise decreases in current amplitude required to induce a plantar flexion were found. These observations are new when specifically looking at LI-NMES via TTE. However, our results are substantiated by earlier studies using higher-intensity NMES, and in line with our primary hypothesis (Baker et al. 1993).

### Strength and limitations

The major strength of this study was applying previously investigated approaches (using standard non-textile electrodes) on the effect of NMES parameter settings on outcomes, into the context of garment-integrated textile electrodes using LI-NMES, by the use of a custom-constructed sock with knitting integrated TTE. The biggest limitation of this study was the low number of participants. However, we consider this to be the first explorative step towards future larger studies focused on improving compliance with NMES.

## Conclusion

Low intensity-NMES applied to the calf via a sock with knitting integrated TTE, is sufficient to produce a clinically relevant muscle activity in the form of plantar flexion of the ankle which may increase blood flow and prevent muscle atrophy. This setup is, however, dependent on frequency and phase duration to reduce discomfort and current amplitude and to improve energy consumption. The best frequencies to minimize discomfort were 1 Hz and 36 Hz. A 400 µs phase duration required the lowest current amplitude. Despite this, phase duration had no significant effect on discomfort. A frequency of 1 Hz and any of the phase durations of 150, 200 or 400 µs were found to be the most energy-efficient options. Further research on LI-NMES via a sock with TTE may in the future help to improve patient compliance to reduce the negative effects of physical inactivity.

## Data Availability

The data generated and/or analyzed during the current study are not publicly available but are available from the corresponding author on reasonable request.

## Abbreviations

IQR: Interquartile range
LI-NMES: Low-intensity neuromuscular electrical stimulation
NMES: Neuromuscular electrical stimulation
NRS: Numerical rating scale
TTE: Transverse textile electrode
VTE: Venous thromboembolism
E_total_: Total energy consumption per NMES stimulation cycle (Joule)
RUT: Ramp-up time (seconds)
RDT: Ramp-down time (seconds)
PT: Plateau time (seconds)
F: Frequency (Hertz)
PD: Pulse duration (= 2 * phase duration) (seconds)
MCA: Max current amplitude (Ampere)
