# Correction to: Neuromuscular electrical stimulation in garments optimized for compliance

**DOI:** 10.1007/s00421-023-05211-6

**Published:** 2023-05-08

**Authors:** R. Juthberg, J. Flodin, L. Guo, S. Rodriguez, N. K. Persson, P. W. Ackermann

**Affiliations:** 1 Department of Molecular Medicine and Surgery, Karolinska Institutet, Stockholm, Sweden; 2 Smart Textiles and Polymeric E-textiles, Swedish School of Textiles, University of Borås, Borås, Sweden; 3 School of Electrical Engineering and Computer Science, KTH Royal Institute of Technology, Stockholm, Sweden; 4 Department of Trauma, Acute Surgery and Orthopaedics, Karolinska University Hospital, Stockholm, Sweden

**Correction to: European Journal of Applied Physiology** 10.1007/s00421-023-05181-9

The original version of this article unfortunately contained a mistake. A small error in the energy-formula.

The original correct formula should be

$$ E_{{{\text{total}}}} = \left( {\frac{{{\text{RUT}} + {\text{RDT}}}}{2} + {\text{PT}}} \right)*F*{\text{PD}}*({\text{MCA}})^{2} *R $$

